# Greater tau pathology is associated with altered predictive coding

**DOI:** 10.1093/braincomms/fcac209

**Published:** 2022-08-17

**Authors:** Klevest Gjini, Cameron Casey, Sean Tanabe, Amber Bo, Margaret Parker, Marissa White, David Kunkel, Richard Lennertz, Robert A Pearce, Tobey Betthauser, Bradley T Christian, Sterling C Johnson, Barbara B Bendlin, Robert D Sanders

**Affiliations:** Department of Neurology, University of Wisconsin, Madison, WI, USA; Department of Anesthesiology, University of Wisconsin, Madison, WI, USA; Department of Anesthesiology, University of Wisconsin, Madison, WI, USA; Department of Anesthesiology, University of Wisconsin, Madison, WI, USA; Department of Anesthesiology, University of Wisconsin, Madison, WI, USA; Department of Anesthesiology, University of Wisconsin, Madison, WI, USA; Department of Anesthesiology, University of Wisconsin, Madison, WI, USA; Department of Anesthesiology, University of Wisconsin, Madison, WI, USA; Department of Anesthesiology, University of Wisconsin, Madison, WI, USA; Department of Medicine, University of Wisconsin, Madison, WI, USA; Department of Medical Physics, University of Wisconsin, Madison, WI, USA; Department of Medicine, University of Wisconsin, Madison, WI, USA; Department of Medicine, University of Wisconsin, Madison, WI, USA; Specialty of Anaesthetics, University of Sydney, Camperdown, Australia; Department of Anaesthetics, Royal Prince Alfred Hospital, Camperdown, Australia; Central Clinical School & NHMRC Clinical Trials Centre, Institute of Academic Surgery, Camperdown, Australia

**Keywords:** predictive coding, tau, mismatch negativity, auditory event-related potentials, dynamic casual modelling

## Abstract

Altered predictive coding may underlie the reduced auditory mismatch negativity amplitude observed in patients with dementia. We hypothesized that accumulating dementia-associated pathologies, including amyloid and tau, lead to disturbed predictions of our sensory environment. This would manifest as increased reliance on ‘observed’ sensory information with an associated increase in feedforward, and decrease in feedback, signalling. To test this hypothesis, we studied a cross-sectional cohort of participants who underwent PET imaging and high-density EEG during an oddball paradigm, and used dynamic casual modelling and Bayesian statistics to make inferences about the neuronal architectures (generators) and mechanisms (effective connectivity) underlying the observed auditory-evoked responses. Amyloid-β imaging with [C-11] Pittsburgh Compound-B PET was qualitatively rated using established criteria. Tau-positive PET scans, with [F-18]MK-6240, were defined by an MK-6240 standardized uptake value ratio positivity threshold at 2 standard deviations above the mean of the Amyloid(–) group in the entorhinal cortex (entorhinal MK-6240 standardized uptake value ratio > 1.27). The cross-sectional cohort included a total of 56 participants [9 and 13 participants in the Tau(+) and Amyloid(+) subgroups, respectively: age interquartile range of (73.50–75.34) and (70.5–75.34) years, 56 and 69% females, respectively; 46 and 43 participants in the Tau(−) and Amyloid(−) subgroups, respectively: age interquartile range of (62.72–72.5) and (62.64–72.48) years, 67 and 65% females, respectively]. Mismatch negativity amplitudes were significantly smaller in Tau+ subgroup than Tau− subgroup (cluster statistics corrected for multiple comparisons: *P* = 0.028). Dynamic causal modelling showed that tau pathology was associated with increased feedforward connectivity and decreased feedback connectivity, with increased excitability of superior temporal gyrus but not inferior frontal regions. This effect on superior temporal gyrus was consistent with the distribution of tau disease on PET in these participants, indicating that the observed differences in mismatch negativity reflect pathological changes evolving in preclinical dementia. Exclusion of participants with diagnosed mild cognitive impairment or dementia did not affect the results. These observational data provide proof of concept that abnormalities in predictive coding may be detected in the preclinical phase of Alzheimer’s disease. This framework also provides a construct to understand how progressive impairments lead to loss of orientation to the sensory world in dementia. Based on our modelling results, plus animal models indicating that Alzheimer’s disease pathologies produce hyperexcitability of higher cortical regions through local disinhibition, mismatch negativity might be a useful monitor to deploy as strategies that target interneuron dysfunction are developed.

## Teaser

Dynamic causal modelling showed that tau pathology was associated with increased feedforward connectivity and decreased feedback connectivity, with increased excitability of superior temporal gyrus but not inferior frontal regions. These data are consistent with preclinical data concerning the changes in neuronal dynamics with dementia pathology.

## Graphical Abstract

**Graphical abstract:**
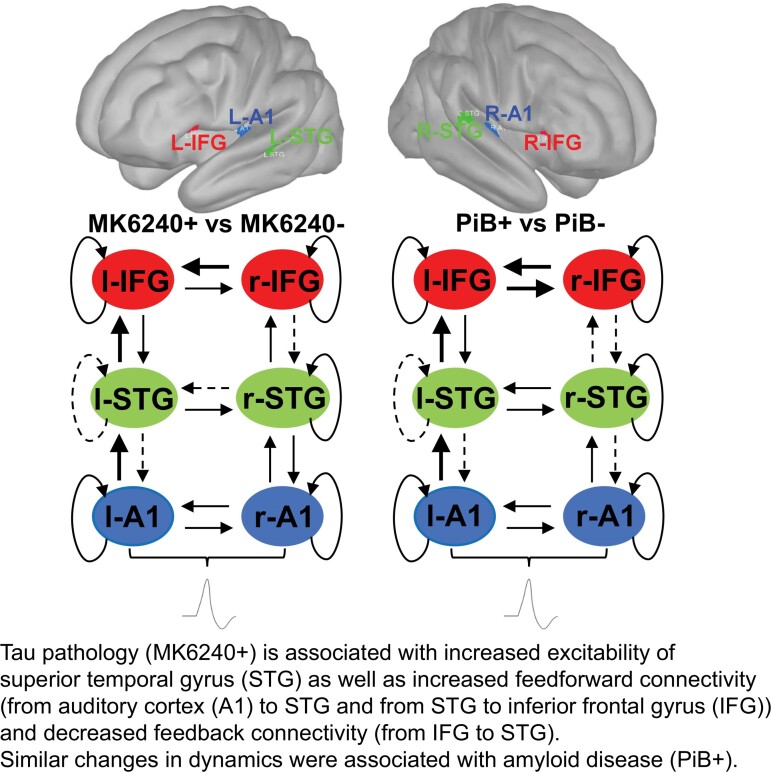

## Introduction

In the later stages of dementia due to Alzheimer’s disease, sufferers lose orientation in time, space and person, which is perhaps the most distressing of the dementia symptoms. There is suggestive evidence that these impairments may start accruing in preclinical stages.^1^ Presently, these disturbing symptoms are largely untreatable when they occur, driving interest in interventions in the preclinical stages of Alzheimer’s disease. Here, we frame this loss of orientation to the sensory environment using the framework of predictive coding,^2^ hypothesizing that within this model, accumulating Alzheimer’s disease pathologies would be associated with disturbance of brain function. In predictive coding, higher order cortical regions constantly make, and update, hypotheses about the sensory environment that are then matched to the actual sensory signals at lower levels of the corticothalamic hierarchy.^2–4^ When there is discordance in the information (i.e. a mismatch in the prediction and the ‘observed’ sensory world), this information is fed up the cortical hierarchy and the higher order prediction is updated. Predictive coding is proposed to provide an explanation for the rapid processing of sensory information as well as how various sensory illusions can occur. Furthermore, predictive coding may explain information processing across a wide range of systems such as sensory, motor and higher order cognitive networks.^3^ However, while theoretically proposed,^4^ associations between predictive coding hypotheses and dementia have not been formally tested. Nor, to our knowledge, has the concept that abnormalities in predictive coding are associated with dementia pathology diagnosed by positron emission tomography (PET).

In dementia, there is already evidence for a breakdown in these predictive coding mechanisms. In particular, a prior study has shown reduced mismatch negativity (MMN) in auditory oddball paradigms.^5^ However, the neural basis for these findings is not yet understood. We hypothesized that accumulating Alzheimer’s disease pathology, namely tau (and amyloid), would be associated with a breakdown in predictive coding. Amyloid accumulates in early disease stages, particularly during the ‘silent’ or asymptomatic phase of Alzheimer’s disease. Tau pathology is considered to accumulate once amyloid burden is present, and level of tau burden correlates with cognitive decline^6^ and increased excitability of neuronal circuits through disinhibition (loss of GABAergic interneuron driven control of signalling).^7,8^ Given that tau pathology represents more advanced disease and more closely linked with network dysfunction, we hypothesized that accumulation of tau would be associated with abnormalities in predictive coding through local disinhibition of higher order cortical regions with impaired generation of predictions,^9^ and hence reduced feedback connectivity,^9^ and an associated increase in feedforward signalling (due to reliance purely on ‘observed’ sensory information, a relatively inefficient method). If proved, this would support models of interneuron dysfunction associated with tau^7^ and mechanisms through which tau may transduce neurodegeneration. As such, this phenotyping may pave the way for identification of individuals who require augmented interneuron function to improve their symptoms.

To test these hypotheses, we employed dynamic causal modelling (DCM) applied to auditory event-related potentials (ERPs) measured with high-density electroencephalography (EEG).^10^ DCM has been used to make inferences about the neuronal architectures that generate the electrophysiological signals in the brain. DCM employs a generative neural mass model describing how the electrophysiological signals are produced at the neuronal level and Bayesian statistics to infer the neuronal mechanisms (discretized as effective connectivity) underlying observed evoked electrophysiological responses.^10^ Using a roving oddball paradigm that controls for changes in the physical features of the stimulus,^11^ DCM has suggested important changes in neuronal connectivity and excitability in pharmacological^12^ and most importantly disease states.^13^

## Materials and methods

Fifty-six participants (>45 years old) were recruited from two on-going cohort studies: 42 participants from the Wisconsin Registry for Alzheimer’s Prevention and 14 participants from the Alzheimer’s Disease Research Center, where brain imaging (PET) data were collected. The inclusion and exclusion criteria are included in the Supplementary Material. The study was approved by the University of Wisconsin-Madison Institutional Review Board and all participants provided informed consent. Participants volunteered for resting state^14^ and roving oddball auditory stimulation collection of EEG data.

### PET imaging analysis

Amyloid-β imaging with [C-11] Pittsburgh Compound-B (PiB) PET employed a dynamic 70-min protocol^15^ and were qualitatively rated using established criteria.^15^ Tau PET imaging, with [F-18]MK-6240, was acquired from ∼70- to 110-min post injection.^16,17^ Tau-positive PET scans were defined by an MK-6240 standardized uptake value ratio (SUVR) positivity threshold at 2 standard deviations above the mean of the PiB(–) group in the entorhinal cortex (entorhinal MK-6240 SUVR > 1.27) as in Betthauser *et al*.^16^).

Out of the total of 56 participants, 9 showed evidence of brain tau pathology (Tau+ subgroup) and 46 without (Tau− subgroup)^16,17^; also 13 were with markers of amyloid beta pathology (PiB+ subgroup) and 43 without (PiB− subgroup)^15^ (Supplementary Fig. 1 and Supplementary Table 1). A further breakdown of demographic and neuropsychological test data according to combined Tau and PiB status is provided in Supplementary Table 2. Five participants had a diagnosis of dementia or mild cognitive impairment (Alzheimer’s disease/minimal cognitive impairment [MCI])). Three of these participants were Tau+/PiB+ and two were Tau−/PiB−. The primary outcome was the difference in the DCM estimates of connectivity between Tau+ and Tau− subjects. As a secondary analysis, we investigated the impact of amyloid positivity on estimates of connectivity.

### The auditory paradigm

**Figure 1:**
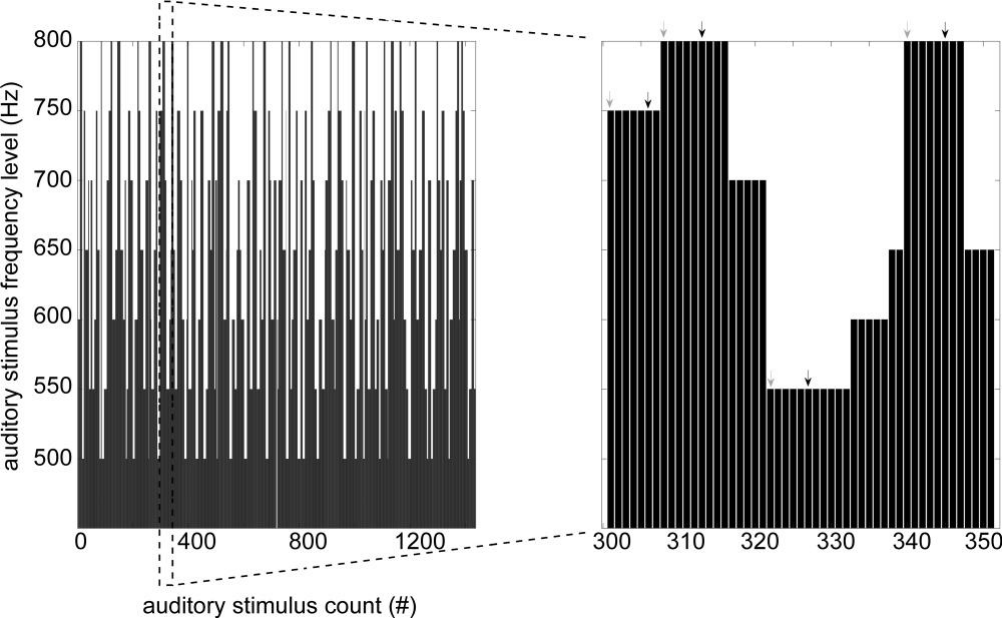
**A schematic display of the auditory roving ‘oddball’ paradigm.** The stimuli were pure sinusoidal tones belonging to seven frequencies varying from 500 to 800 Hz in steps of 50 Hz. They were presented in a sequence, with a roving, or sporadically changing tone. The duration of each tone was 70 ms (with 5 ms rise and fall times), and the interstimulus interval was set to 500 ms. The deviant (first tone in a train of at least six tones of the same frequency) and standard stimuli (sixth tone in the same train) are marked with grey and black arrows, respectively.

The auditory roving ‘oddball’ paradigm (adapted from Garrido *et al*.^18^) consisted in the presentation of a sequence of pure sinusoidal tones, with a sporadically changing tone (Fig. 1). Each stimulus train was comprised of tones of one frequency followed by a train of tones of different frequencies. Deviants and standards had the same physical properties given that the first tone of a train was a deviant and became a standard after few repetitions. The sequence of the auditory stimuli was delivered via a stimulus presentation software (E-Prime; Psychology Software Tools, Inc., Pittsburgh, PA, USA). The subjects were awake and monitored during the presentation of the auditory paradigm. They were not required to focus their attention or perform any particular task as this could potentially lead to biases related to how well subjects could attend to a task and retain information. As such instructions were not given to attend to any tone in particular or to complete a task.

### EEG data acquisition, preprocessing and extraction of ERPs

High-density EEG data were acquired with a 250 Hz sampling rate using a 256-channel system (Electrical Geodesics, Inc., Eugene, OR, USA), capable of accepting eight-bit digital trigger input (i.e. TTL pulses). EEG data were processed in EEGLAB (https://sccn.ucsd.edu/eeglab/), a toolbox running in MATLAB (https://www.mathworks.com/) and its plugin ERPLAB (https://erpinfo.org/erplab). EEGs were bandpass filtered between 1 and 30 Hz, data segments heavily compromised by artefacts were detected by visual inspection and then removed (a concurrent display of 10 s windows of data from all sensors), the detected noisy channels were rejected and re-interpolated and non-neuronal artefactual components (due to eye movements, muscle activity and cardiac electric field) were detected and rejected using independent component analysis (ICA). More specifically, the applied ICA-based artefact detection and rejection do not reject whole segments of data, only remove the ECG artefact (or any other type of identified physiological artefact) contamination to the data leaving the respective data length intact. Then EEG data were segmented to epochs of interest [(−100 to 400 ms) from stimulus onset at 0 ms] separately for deviant (first stimulus in a train of at least six tones of the same frequency) and standard stimuli (sixth stimulus in the trains with same characteristics as mentioned for deviants), and bad epochs were manually flagged for rejection. Finally, in each individual subject the auditory ERPs for standard and deviant stimuli were obtained by the method of averaging of the previously obtained epochs followed by baseline correction [i.e. subtraction of the mean amplitude during the baseline (−100 to 0 ms) interval]. Difference waves were calculated by subtracting the averaged ERP of standard stimuli from the averaged ERP to deviant stimuli. MMN amplitudes were obtained from the difference waves at each time point as well as mean scores at intervals of interest post-stimulus onset [(148–160 ms) and (148–200 ms)]. Grand averages for ERPs of standard, deviant and MMN waveforms in Tau+, Tau−, PiB+ and PiB− subject subgroups were obtained by averaging respective individual subject ERPs. No significant differences were found in the accepted number of trials for standard and deviant stimuli between the Tau+ and Tau− subgroups (independent samples *t*-test: *P* > 0.05).

### Statistics on scalp ERP data

First-level nonparametric *t*-statistics between the MMN amplitudes from the compared subgroups (Tau+ versus Tau−, PiB+ versus PiB−) at each sensor of the 256-channel array (at individual time points and averages from time intervals of interest) were followed by second-level spatiotemporal cluster-based statistics were carried out in FieldTrip toolbox (https://www.fieldtriptoolbox.org/) running in MATLAB. The second-level test statistic (i.e. the maximum of the cluster-level summed *t*-values) is calculated on the observed experimental partition and on large number (5000) of random partitions. The resulting Monte Carlo significance probability, which is also called in this case a *P*-value, is corrected for ‘multiple comparisons’. The significance at both levels was set at *P* < 0.05.

### Dynamic casual modelling and Bayesian model comparison

DCM module for ERPs in SPM12 (https://www.fil.ion.ucl.ac.uk/spm) is used to estimate effective connectivity between brain regions and test the effect of experimental perturbations on coupling among the involved sources generating the acquired ERP signals. The DCM general methodology when applied to ERP data supplements the conventional electromagnetic forward models with a model explaining how source activity is generated by neuronal dynamics, and enables inference about both the spatial involvement of sources and the underlying neuronal architecture generating the signals.^10^ The selection of the involved brain areas as part of a given task are based on previous studies reported in the literature.

DCM models: Eighteen DCMs (M1-M18) were used for Bayesian model comparison. Each model receives (parameterized) subcortical input at the A1 sources, which elicit transient perturbations in the remaining sources. M1–M11 were identical to those run in Boly *et al*.^13^ Further extensions in Models M13–M18 were based on network level work on generation of auditory MMN.^19^ The 18 different models are fitted to the individual data from the whole set of subjects and subgroups separately in order to obtain estimates of the parameters. Different DCM models include different numbers of sources (i.e. two, four, five, six). The models were generated using the entire post-stimulus window (0–400 ms) setting the prior for when the auditory cue is supposed to arrive at cortex to 60 ms based on literature recommendations on what was used in previous DCM papers involving auditory oddball paradigms.

The random-effects Bayesian model selection was used to test which population-level best/winning model had the greatest evidence. The winning model (M18) was selected for subsequent quantitative analysis of effective connectivity across the two populations studied (Tau+ versus Tau− subgroup; PiB+ versus PiB− subgroup).

### Parametric empirical Bayes

Parametric empirical Bayes (PEB) analyses (based on the ‘B’ modulation matrix in DCM module of SPM12) were done only with DCM data from the winning model (M18). The PEB model has parameters encoding the deviation from the mean due to the group difference. For the group difference, positive estimated parameters indicate stronger connectivity in (+) Tau or PiB group than (−) Tau or PiB group and negative parameters indicate the opposite. The obtained posterior probabilities >95% correspond to a strong evidence level for the effect of interest.

### Sample size

An *a priori* sample size was not conducted, as these data were collected as part of a study that also collected resting state EEG that is already published.^14^ All available data from the cohort study were included in this work.

#### Data availability

Data are available from the authors on reasonable request and adhered to local ethics.

## Results

### Sensor ERP data

**Figure 2:**
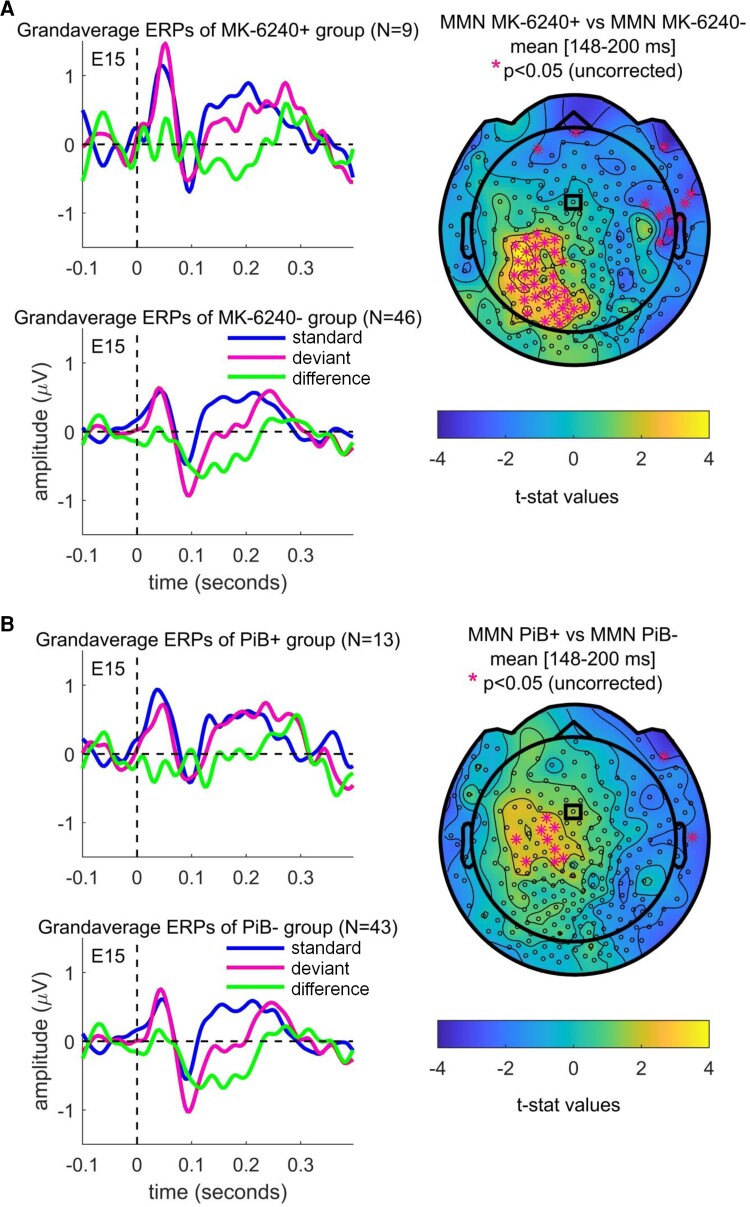
**Grandaverage ERPs and MMN topographies.** (**A**) Display of grandaverage ERPs for Tau+ (MK-6240+) and Tau− (MK-6240−) participants (standard, deviant and difference waves) from a representative sensor (E15/Fz) highlighted with the small black rectangle in the topoplot, and topographical plots of independent samples *t*-statistics values for comparison of the mean MMN amplitudes between MK-6240+ and MK-6240− participants for the time interval (148–200 ms) from stimulus presentation onset. (**B**) The same shown for data from amyloid scanning (PiB+ and PiB−) participants. In the topoplots, sensors showing significant differences (*P* < 0.05, uncorrected for multiple comparisons) in the mentioned interval mean MMNs between (+) and (−) subjects in each of the evaluated time intervals are highlighted with magenta asterisks.

At sensor level, MMN amplitudes were found to be significantly smaller in the Tau+ subgroup than the Tau− subgroup. Figure 2A displays grandaverage ERPs for Tau+ and Tau− subject groups (overlaying the standard, deviant and difference waves) from a representative sensor (E15/Fz), and shows topographical plots of first-level nonparametric permutation statistics comparing the mean MMN for time interval (148–200 ms) from stimulus presentation onset between Tau+ and Tau− subject groups; the same is shown for PiB+ and PiB− participant groups in Fig. 2B. The sensors showing significant differences (*P* < 0.05, uncorrected for multiple comparisons) in mean MMNs between (+) and (−) subjects in the evaluated time interval are highlighted with magenta asterisks.

**Figure 3:**
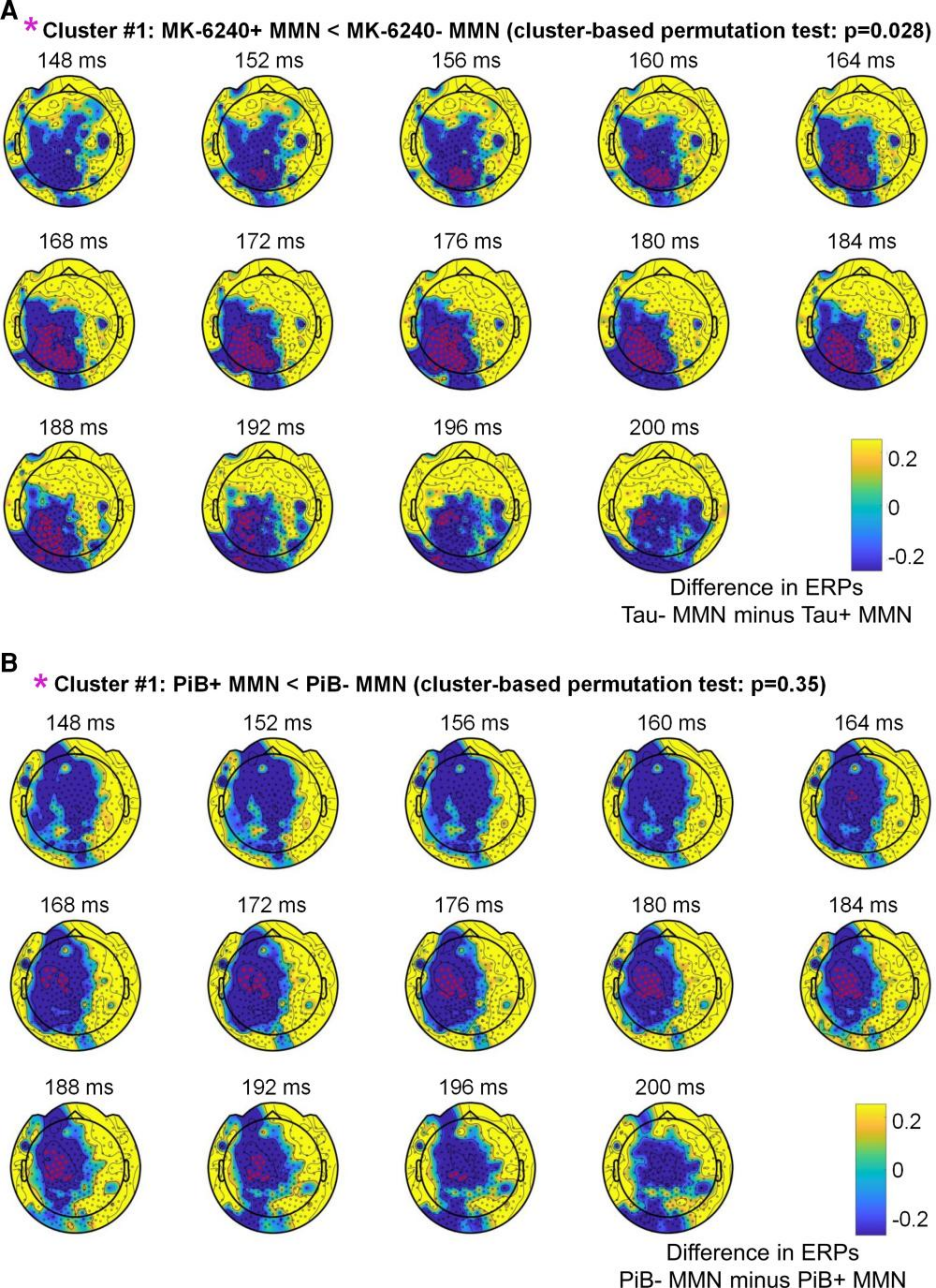
**Statistical comparison of MMNs (Tau+ versus Tau−; PiB+ versus PiB−).** (**A**) Display of the second-level cluster-based permutation statistics (correcting for multiple comparisons) for comparison of mean MMNs between Tau+ (MK-6240+) and Tau− (MK-6240−) participants, and (**B**) amyloid scanning (PiB+ and PiB−) participants. Sensors belonging to the cluster with the largest differences in MMN between (+) and (−) subjects are highlighted with magenta asterisks.

The second-level spatiotemporal cluster-based permutation statistics (correcting for multiple comparisons) of mean MMNs between Tau+ and Tau− participants during the interval of (148–200 ms) showed a statistically significant difference (*P* = 0.028), with sensors belonging to the cluster with the largest differences in MMN between (+) and (−) participants highlighted with magenta asterisks in Fig. 3A. As a secondary analysis, we conducted a similar analysis for amyloid disease. No significant differences were found for the same comparison of mean MMNs between PiB+ and PiB− groups (Fig. 3B). Additional information on scalp ERPs is provided in Supplementary Figs 2–5.

### DCM, Bayesian model comparison and PEB analysis

The random-effects Bayesian model selection method showed that the fully connected model (M18) had the greatest evidence and this model was selected for subsequent quantitative analysis of effective connectivity between the respective subgroups with positive (+) or negative (−) presence of pathology biomarkers. Furthermore, the family-wise Bayesian model selection procedure, in the two populations studied, showed that the best models included two frontal regions (bilateral) and the presence of both backward and forward connections, as well as lateral connections. Finally, the random-effects Bayesian model selection showed that a model representing a fully connected original M18 model with modulation of intrinsic/self-connection at each node had greater evidence compared to the original M18 model without modulation of intrinsic/self-connection at each node, and the resulting winning model was selected for subsequent quantitative analysis of effective connectivity across the two populations (Supplementary Fig. 7A–C).

**Figure 4:**
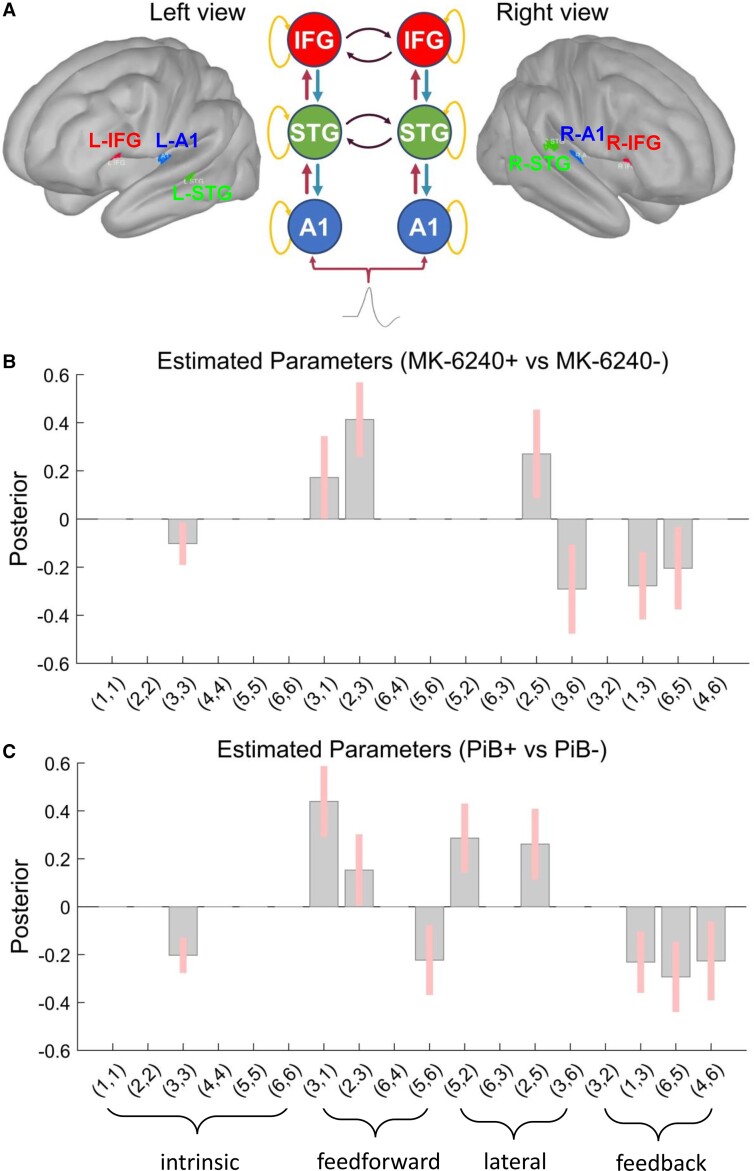
**PEB modelling results.** Display of the results of the second-level PEB analysis following the DCM-based estimation (i.e. fitting the Winning Model **A** to the individual data, to get estimates of the parameters). The PEB model has parameters encoding the deviation from the mean due to the group difference (Covariate 2). For the group difference, positive estimated parameters indicate stronger connectivity in (+) Tau (MK-6240) or PiB group than (−) Tau or PiB group and negative parameters indicate the opposite. Posterior probabilities >95% (corresponding to a strong evidence level) for the deviance detection effect of interest are shown. (**A**) Sources in the left view of the cortex as (1) left A1—in blue; (2) left IFG—in red; (3) left STG—in green; sources in the right view of the brain as (4) right A2—in blue; (5) right IFG—in red; (6) right STG—in green. The schematic view of the winning model M18 (including modulation of intrinsic connections) displays the intrinsic, feedforward, lateral and feedback connections with orange, red, black and blue curved line arrows, respectively. (**B**) PEB [Tau+ MMN versus Tau− MMN] results interpretation (increase/decrease in connection strength): (3,3) left STG (decreased self-inhibition in Tau+); (3,1) left A1 → left STG (increased in Tau+); (2,3) left STG → left IFG (increased in Tau+); (2,5) right IFG → left IFG (increased in Tau+); (3,6) right STG → left STG (decreased in Tau+); (1,3) left STG → left A1 (decreased in Tau+); (6,5) right IFG → right STG (decreased in Tau+). (**C**) PEB (PiB+ MMN versus PiB− MMN) results interpretation (increase/decrease in connection strength): (3,3) left STG (decreased self-inhibition in PiB+); (3,1) left A1 → left STG (increased in PiB+); (2,3) left STG → left IFG (increased in PiB+); (5,6) right STG → right IFG (decreased in PiB+); (5,2) left IFG → right IFG (increased in PiB+); (2,5) right IFG → left IFG (increased in PiB+); (1,3) left STG → left A1 (decreased in PiB+); (6,5) right IFG → right STG (decreased in PiB+); (4,6) right STG → right A1 (decreased in PiB+). Connections: (*x*, *y*) are interpreted as *y* → *x*.

Results from PEB analysis (done only with DCM data from the ‘winning’ fully connected M18 model including modulation of intrinsic/self-connection at each node; Fig. 4A) showed significant increases/decreases in connection strength in group differences of the deviant stimulus effect between Tau+ and Tau− subjects (Fig. 4B). Results show increased feedforward connectivity in tau pathology with impaired feedback connectivity and increased excitability of superior temporal gyrus, but not inferior frontal regions. As a secondary analysis, we showed significant increases/decreases in connection strength in group differences of the deviant stimulus effect between PiB+ and PiB− groups as well (Fig. 4C).

Supplementary Fig. 8A and B displays the grandaveraged source waveforms during the interval (0–400 ms) for standard, deviant and difference waves from data belonging to Tau+ and Tau−, and PiB+ and PiB− groups, respectively.

## Discussion

These data suggest that tau pathology is associated with changes in the auditory-evoked response, indicative of impairments of predictive coding, with associated alterations in hierarchical connectivity and regional excitability. Of note, these data are consistent with prior studies of dementia focusing on scalp EEG.^20^ They also suggest that the predictive coding framework may provide a useful construct for understanding dementia pathogenesis in the preclinical phase, particularly through identification of changes in neuronal excitation and connectivity. As amyloid pathology is a defining feature of Alzheimer’s disease dementia and is known to disturb cellular excitability, secondary analyses examined the effect of amyloid on predictive coding. It is important to note that in our secondary analysis, we did not find robust scalp level changes related to amyloid disease in this study, but did find some similar changes in effective connectivity, suggesting that in this earlier stage of disease, there may be some functional compensation. Our scalp level sensitivity analyses did support the notion that the signal was not entirely driven by symptomatic individuals with MCI/Alzheimer’s disease but rather reflects associations with Alzheimer’s disease pathology. In turn, an inference is that accumulating pathology leads to an alteration in predictive coding prior to substantial changes in cognition. However, we note that the current sample was small, so we propose that larger studies in preclinical Alzheimer’s disease are needed to confirm this. While, by definition, we were not underpowered to identify significant effects in this cohort, future larger studies are required to validate and identify the generalizability of our findings.

Prior studies have linked dementia to disabled predictive coding via impairments in MMN,^5^ with theoretical arguments advanced for why predictive coding is a useful framework for understanding dementia.^4^ To our knowledge studies have not linked *in situ* pathologies in humans to these abnormalities, though it is established that dementia sufferers exhibit reduced MMN in oddball paradigms similar to the one we have used. The demonstration that there is increased excitability of superior temporal gyrus, a higher order cortical region, is consistent with animal evidence from tau and amyloid disease models^21^ and as a possible mechanism of excitotoxic neurodegeneration. We have previously identified tau disease in temporal lobe, but not inferior frontal regions, in our subjects.^16^ Hence, our DCM results showing increased excitability of temporal, but not frontal, regions are consistent with *in situ* tau pathologies disturbing cellular excitability. This provides further plausibility for our results.

Our results of increased feedforward connectivity, with impaired feedback connectivity, are consistent with our *a priori* hypotheses. In essence, this represents a shift from predictive coding models of sensory perception to more ‘classical’ feedforward models.^2^ A proposed disadvantage of these classical models is their relative inefficiency of information processing. A shift to a more inefficient mechanism of information processing would be consistent with cognitive failure and progressive disorientation to the sensory world. Our data are also consistent with various animal models suggesting a critical role of interneuron dysfunction in dementia pathogenesis^7,8^ and provide impetus to developing selective therapies in that domain.

We note some important limitations of our work. These data are observational and, due to the small sample size, they can be considered proof of concept, but not definitive. Future validation in a larger cohort is warranted. Similarly, future studies should look for a biological gradient between changes in excitability and connectivity of regions and absolute load of disease as quantified in PET. Corresponding studies in animals would also be beneficial to help establish causality. It is also unclear why we observed predominantly left hemispheric effects on connectivity, this may be due to a combination of some or all of: (i) predominant left hemispheric disease or metabolic failure at the disease stages we study,^22^ (ii) different signal to noise ratios in detecting EEG changes for left and right hemispheres and (iii) the small sample size. Future research, with larger sample sizes, should investigate if PET changes are only associated with left hemispheric changes, whether this is merely a nuance of our data set or whether this reflects the underlying disease at difference stages of disease progression. Ultimately, while this work uses established modelling techniques to estimate changes in neuronal function, only interventional studies, perhaps targeting interneurons who critically regulate local excitation and feedforward and feedback connectivity, can establish the underlying mechanisms. Designing those studies and developing therapies targeting interneurons is a significant challenge and hence these in-human data supporting such an ambitious task are critical to the field.

## Supplementary Material

fcac209_Supplementary_Data

## Abbreviations

DCM =: dynamic causal modelling
MMN =: mismatch negativity
PiB =: Pittsburgh compound B
SUVR =: standardized uptake value ratio
