# Supplementary material for: Greater tau pathology is associated with altered predictive coding
Source: Brain Commun. 2022 Aug 17;4(5):fcac209. doi: 10.1093/braincomms/fcac209 (PMC9547525; doi:10.1093/braincomms/fcac209)
Supplement: fcac209_Supplementary_Data [file fcac209_supplementary_data.pdf]

Inclusion/Exclusion Criteria for EEG-AD:

A. Age >18 years old

B. Current enrollment or previous participation in a research study with ADRC or WRAP where brain imaging (i.e. PET, MRI) data has been collected.

Exclusion criteria

A. Individuals lacking the capacity to provide informed consent, as determined by a clinician.

Wisconsin ADRC Clinical Core Inclusion/Exclusion Criteria

Six subgroups of participants will be enrolled in the **Wisconsin ADRC Clinical Core** (referred to hereafter as the Clinical Core):

1) Individuals with mild late-onset Alzheimer's disease (referred to hereafter as AD)
2) Individuals with Mild Cognitive Impairment (referred to hereafter as MCI)
3) Age-matched healthy older controls
4) Middle-aged adults with a parental history of AD: IMPACT cohort (parental history positive)
5) Middle-aged adults whose father survived to 70 and mother 75 without signs or symptoms of dementia: IMPACT controls (parental history negative)
6) Middle-aged adults with indeterminate family history of AD (i.e. parent did not live to age 70; participant is adopted; parent has MCI, but not AD): IMPACT indeterminate category

The latter three groups are designated IMPACT parental history positive, IMPACT parental history negative, and IMPACT Indeterminate categories, respectively (**I**nvestigating **M**emory in **P**reclinical **A**D - **C**auses and **T**reatments). **Tables 1** through **4** list specific inclusion and exclusion criteria for participant groups.

Eligibility Criteria for AD Participants

Inclusion Criteria:

- Age ≥45 years
- Meets NINCDS-ADRDA criteria for probable AD
- Retains decisional capacity at initial visit
- Ability to fast from food and drink for 12 hours

Exclusion Criteria:

- Severe kidney dysfunction requiring hemodialysis
- Severe congestive heart failure
- History of clinically significant ischemic or hemorrhagic stroke, or significant cerebrovascular disease at discretion of investigators
- History of HIV/AIDS
- Severe untreated obstructive sleep apnea
- Major neurologic disorders other than dementia (e.g., MS, ALS, brain surgery, etc. at discretion of investigators)
- Current or recent (<1 year) major psychiatric condition (Axis I) or addictive disorders
- Lack of study partner
- Contraindication to biomarker procedures at baseline visit, unless subject to Table 5 exception
- Other significant medical conditions at investigators' discretion

Eligibility Criteria for MCI Participants

Inclusion Criteria:

- Age ≥45 years
- Meets NIA-AA criteria for MCI
- Retains decisional capacity at initial visit
- Ability to fast from food and drink for 12 hours
- If enrolled under HS IRB # 2015-0030 and not a member of an underrepresented group in research, must agree to participate in biennial biomarker procedures (Biomarker procedures remain optional for those enrolled under HS IRB # H-2009-0103 and underrepresented groups in research).

Exclusion Criteria:

- Severe kidney dysfunction requiring hemodialysis
- Severe congestive heart failure
- History of clinically significant ischemic or hemorrhagic stroke or significant cerebrovascular disease at discretion of investigators
- History of HIV/AIDS
- Severe untreated obstructive sleep apnea
- Major neurologic disorders other than dementia (e.g., MS, ALS, brain surgery, etc. at discretion of investigators)
- Current or recent (<1 year) major psychiatric condition (Axis I) or addictive disorders
- Lack of study partner
- Contraindication to biomarker procedures at baseline visit, unless subject to Table 5 exception
- Other significant medical conditions at investigators' discretion

Eligibility Criteria for Healthy Older Controls

Inclusion Criteria:

- Age >65 years
- Ability to fast from food and drink for 12 hours
- If enrolled under HS IRB # 2015-0030 and not a member of an underrepresented group in research, must agree to participate in biennial biomarker procedures (Biomarker procedures remain optional for those enrolled under HS IRB # H-2009-0103 and underrepresented groups in research).

Exclusion Criteria:

- Severe kidney dysfunction requiring hemodialysis
- Severe congestive heart failure
- History of clinically significant ischemic or hemorrhagic stroke or significant cerebrovascular disease at discretion of investigators
- History of HIV/AIDS
- Severe untreated obstructive sleep apnea
- Major neurologic disorders other than dementia (e.g., MS, ALS, brain surgery, etc. at discretion of investigators)
- Lack of study partner
- Current or recent (<1 year) major psychiatric condition (Axis I) or addictive disorders
- Lack of study partner
- Contraindication to biomarker procedures at baseline visit, unless subject to Table 5 exception
- Other significant medical conditions at investigators' discretion

Eligibility Criteria for IMPACT Parental History Positive, IMPACT Parental History Negative, and IMPACT Indeterminate

Inclusion Criteria:

- Age ≥45 years and ≤65 years
- Biological parent with probable or definite AD (IMPACT cohort)

OR

- Biological mother lived to at least 75 years and father to at least 70 years without symptoms of dementia (IMPACT control)

OR

- Parental history of dementia indeterminate (i.e., parent did not live to above age limits, but did not show signs of dementia; parent has/had MCI; participant is adopted and biological family history is not known; etc.) (IMPACT family history indeterminate)
- Ability to fast from food and drink for 12 hours
- If enrolled under HS IRB # 2015-0030 and not a member of an underrepresented group in research, must agree to participate in biennial biomarker procedures (Biomarker procedures remain optional for those enrolled under HS IRB # H-2009-0103 and underrepresented groups in research).

Exclusion Criteria:

- Enrolled in Wisconsin Registry for Alzheimer's Prevention (WRAP, H-2001-329, H-2006-0202, 2011-0622, 2016-0634)
- Diagnosis of MCI or AD at initial visit (will then be included in other ADRC groups)
- Severe kidney injury requiring hemodialysis
- Severe congestive heart failure
- History of clinically significant ischemic or hemorrhagic stroke, or significant cerebrovascular disease at discretion of investigators
- History of HIV/AIDS
- Severe untreated obstructive sleep apnea
- Major neurologic disorders other than dementia (e.g., MS, ALS, brain surgery, etc. at discretion of investigators)
- Current or recent (<1 year) major psychiatric condition (Axis I) or addictive disorders
- Lack of study partner
- Contraindication to biomarker procedures at baseline visit, unless subject to Table 5 exception
- Other significant medical conditions at investigators' discretion

WRAP Inclusion/Exclusion Criteria

Participants will meet the following inclusion criteria at study entry, though exceptions may be made by the PI or other qualified co-investigator designated by the PI:

- Aged 40-65 at study entry
- Have at least one biological parent diagnosed with dementia due to AD; OR, neither biological parent with a diagnosis of AD (mother lived to 75 years and father lived to 70 years without AD) OR approved by the PI (or other qualified co-investigator designated by the PI) to enroll with indeterminate family history status.
- Ability to speak English fluently and ability to understand and be willing to undergo study procedures for the entire length of the study.
- Visual and auditory acuity adequate for neuropsychological testing.
- Good general health with no diseases expected to interfere with the study.
- A spouse, friend or family member that knows the participant well, that can answer informant-based questionnaires and interviews.

Exclusion Criteria

The following exclusion criteria apply at study entry. Exceptions may be made by the PI, or other qualified co-investigator as designated by the PI, on a case by case basis.

- History or diagnosis of a significant learning disability that may interfere with cognitive assessment or obfuscate a cognitive trajectory.
- Major neurological disorders: A prior diagnosis of dementia or MCI; a proximal diagnosis in adulthood of other major neurological disease such as other neurodegenerative disorder; multiple sclerosis, meningitis with focal sequelae or lingering cognitive deficits, clinically significant cerebrovascular accident, significant traumatic brain injury with loss of consciousness of 60 min or more; brain infection, neoplasm, or neurosurgery.
- Persons with certain cancers in or near the brain whose treatment involved radiation to or near the brain, or chemotherapy within the past 5 years are excluded at entry. These treatments may impact accurate measure of cognitive changes due to aging and or intrinsic AD.
- Current major Axis I DSM-V disorder that may impact accurate measurement of cognition or obfuscate the effect of aging or AD on cognitive change over time. Examples include bipolar disorder, schizophrenia or other psychotic disorder, severe major depression, self-reported drug or alcohol abuse or dependence that, in the opinion of the study PI or a study clinician may interfere with accurate measurement of cognitive change due to aging or AD.
- Lacks capacity to provide informed consent.
- Active participation in another longitudinal study involving cognition that may interfere with valid cognitive measurement in this study.

Inclusion criteria for follow up visits

- Willing and able to continue participation in person in this longitudinal study. A reduced battery of testing and procedures is allowable if the participant is not able or willing to complete the full battery.

- Or, if not willing to return for in-person visits, willing to complete mail-in questionnaires and telephone assessments with the participant and informant.

Exclusion criteria for follow up visits

- Participant has been diagnosed with dementia. This is a stopping point for in-person visits. A final ending visit may be administered. Participants may continue to be followed with forms and phone contact and may be approached for linked studies, including the ADRC clinical core, and the Wisconsin Brain Donor Program.

- Participant has developed medical illness such that cognitive testing is not feasible, would not be valid if completed, or would otherwise interfere with the goals of the study. Examples include post-enrollment acquired brain injury such as significant stroke or traumatic brain injury, movement disorder or mental illness. Such exclusion decisions will be evaluated individually by the study team. A final in person ending visit may be administered.

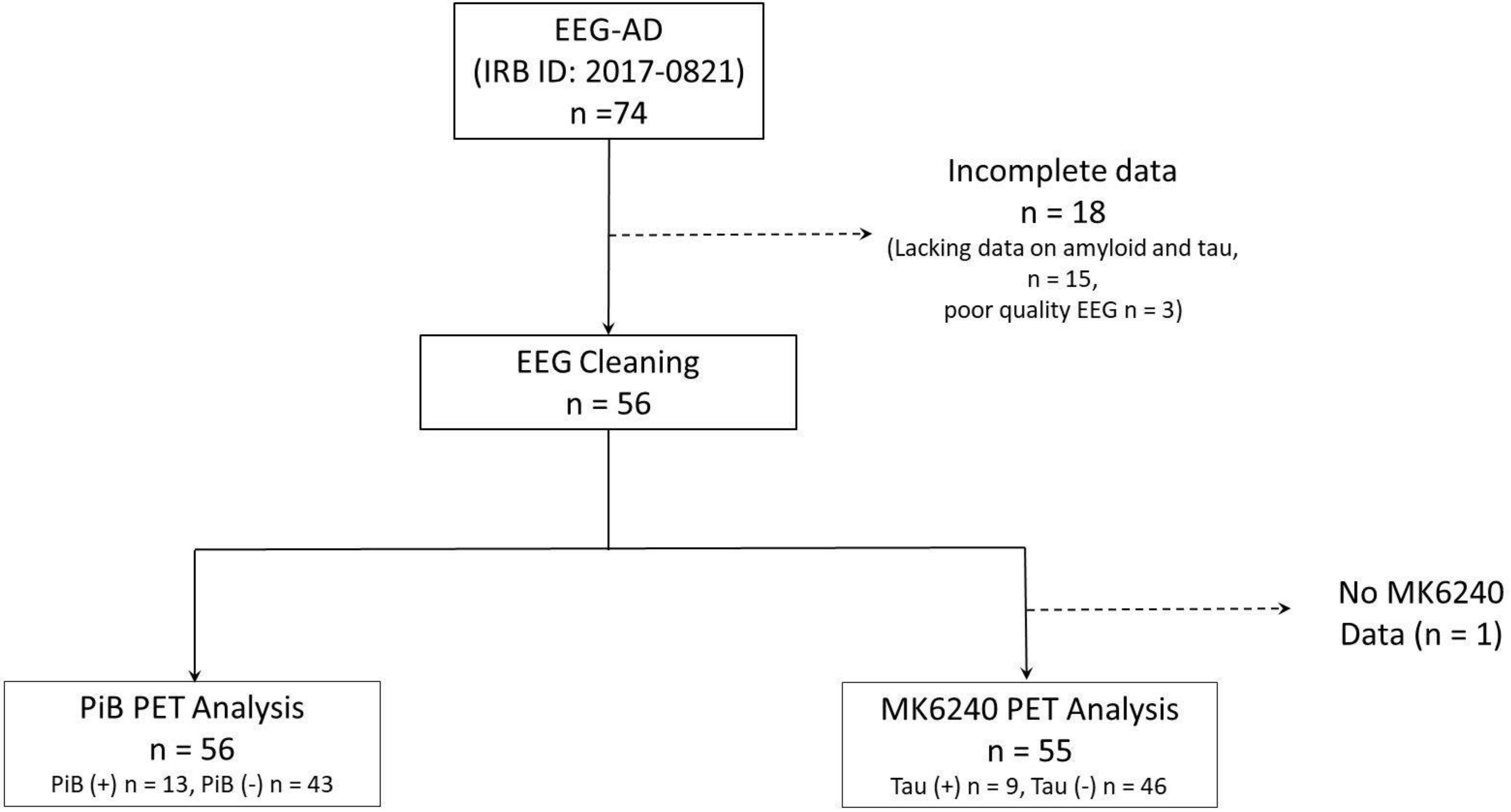

Supplementary Figure 1: Strengthening the Reporting of Observational Studies in Epidemiology (STROBE) diagram.

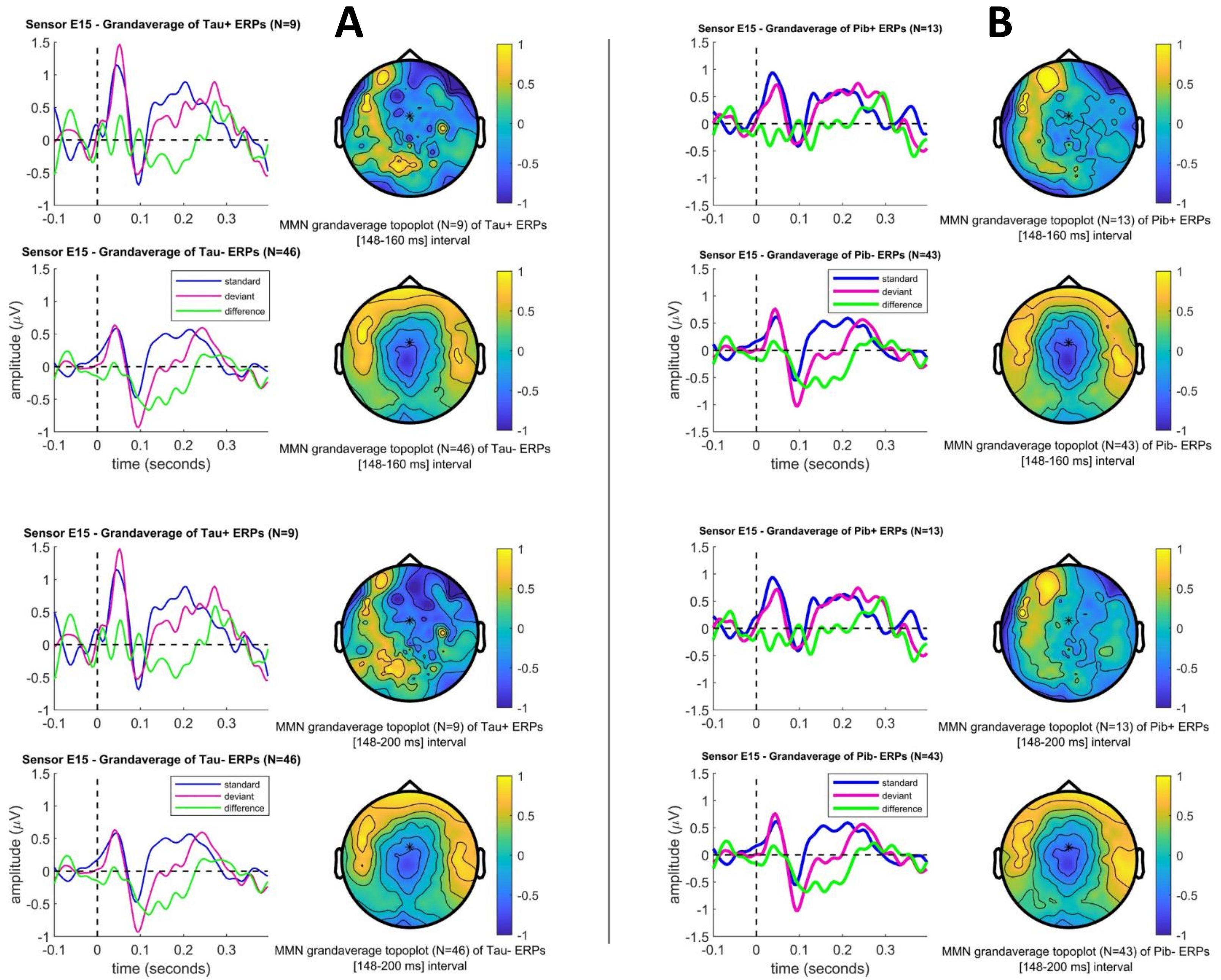

Supplementary Figure 2: In the A section, topographical plots of MMN grandaverages from MK-6240+ and MK-6240- (as Tau+ and Tau-) subjects for two time intervals ([148-160 ms] and [148-200 ms]) are shown, as well as MMN grandaveraged waveforms from a selected sensor E15/Fz highlighted with an asterisk (*) in the displayed topoplots. In the B section the same is displayed for MMN data from PiB+ and PiB- subjects.

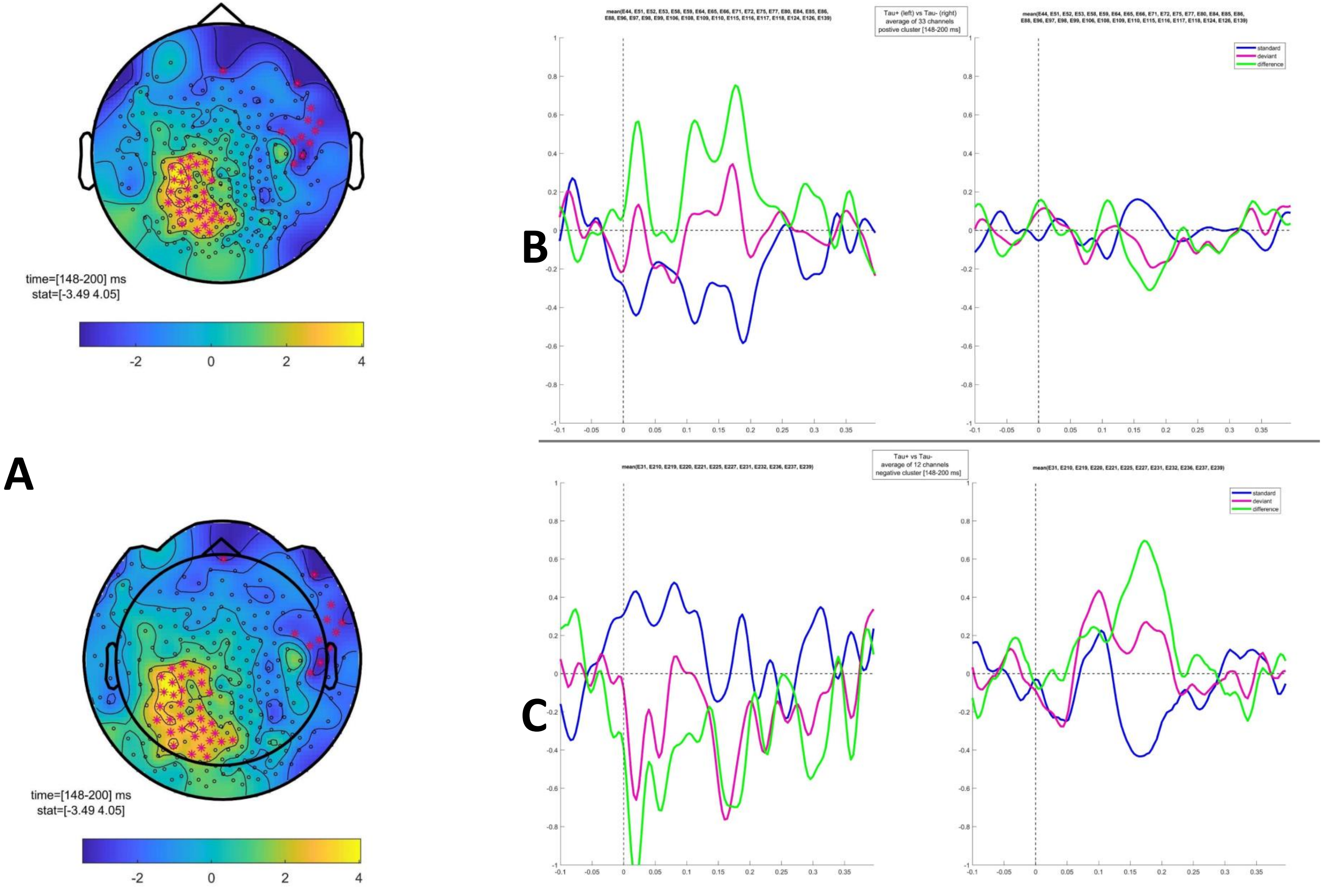

Supplementary Figure 3: In the A section, topographical plots of independent samples t-statistics between MMN mean amplitudes of time interval [148-200 ms] from MK-6240+ and MK-6240-(as Tau+ and Tau-) subjects are shown in two versions (with a circular and a helmet shaped displays). Sensors showing significant differences *(*based on nonparametric permutation statistics*)* in interval mean MMN amplitudes between groups are highlighted with red asterisks. In the B section, average MMN waveforms from the “positive t-stat sensors” (MMN being smaller, i.e. more positive values in MK-6240+ group [left] compared to MK-6240- group [right]) are shown in green. In the C section, average MMN waveforms from the “negative t-stat sensors” (MMN being larger, i.e. more negative values in MK-6240+ group [left] compared to MK-6240- group [right]) are shown in green. ERP waveforms to standard and deviant stimuli are shown in blue and magenta, respectively.

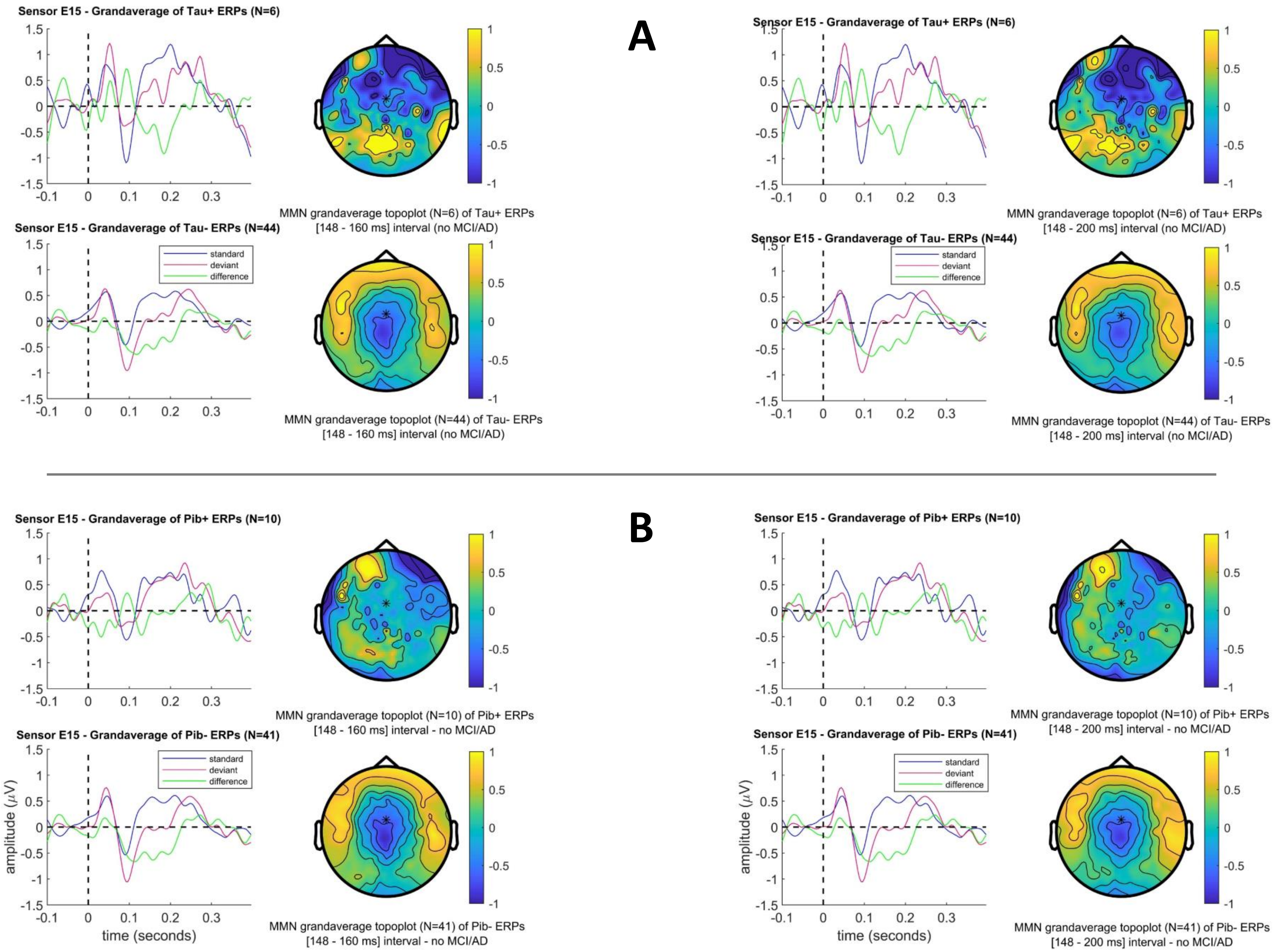

Supplementary Figure 4: (A) Grandaverage ERPs for MK-6240+ and MK-6240- (as Tau+ and Tau-) subjects from a representative sensor (E15 / Fz) highlighted with an asterisk (*) in the displayed topoplots, and topographical plots of the mean MMN of Tau+ and Tau- subjects for two time intervals ([148-160 ms] and [148-200 ms] from stimulus presentation onset); (B) The same shown for data from PiB+ and PiB- subjects. Note: 3 subjects with MCI/AD were removed from the (+) MK-6240/PiB groups, and 2 subjects with MCI/AD were removed from the (-) MK-6240/PiB groups.

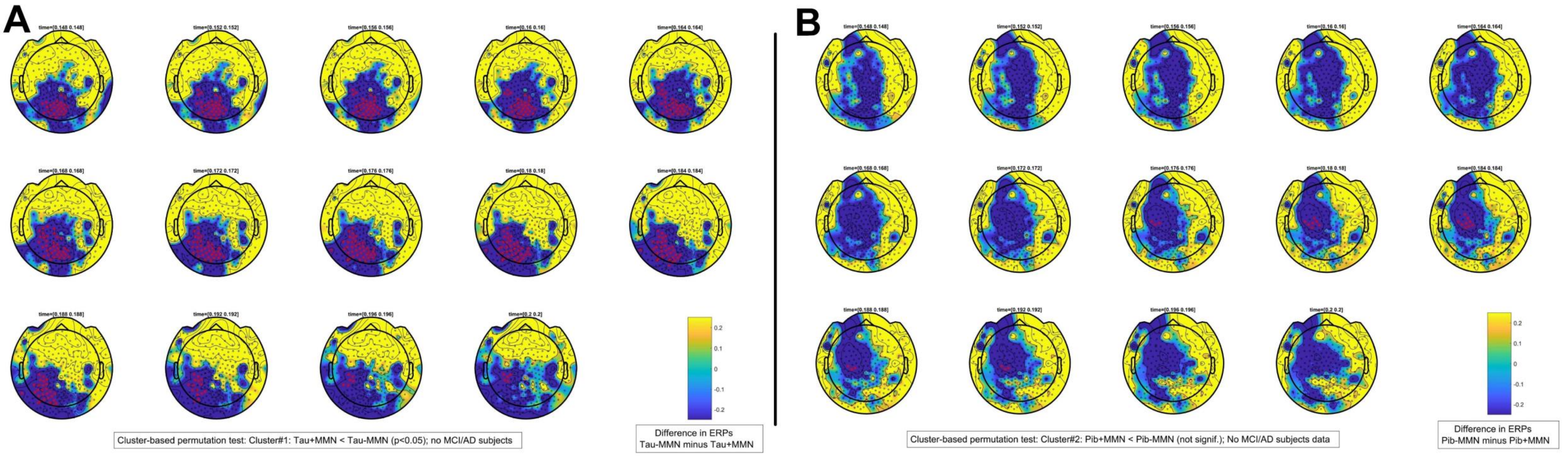

Supplementary Figure 5: (A) Grandaverage ERPs for Tau+ and Tau- subjects (standard, deviant, and difference waves) from a representative sensor (E15 / Fz) highlighted with an asterisk (*) in the displayed topoplots, and topographical plots of the mean MMN of MK-6240+ and MK-6240- (as Tau+ and Tau-) subjects for two time intervals ([148-160 ms] and [148-200 ms] from stimulus presentation onset); (B) The same shown for data from PiB+ and PiB- subjects. Note: 3 subjects with MCI/AD were removed from the (+) MK-6240 /PiB groups, and 2 subjects with MCI/AD were removed from the (-) MK-6240 /PiB groups.

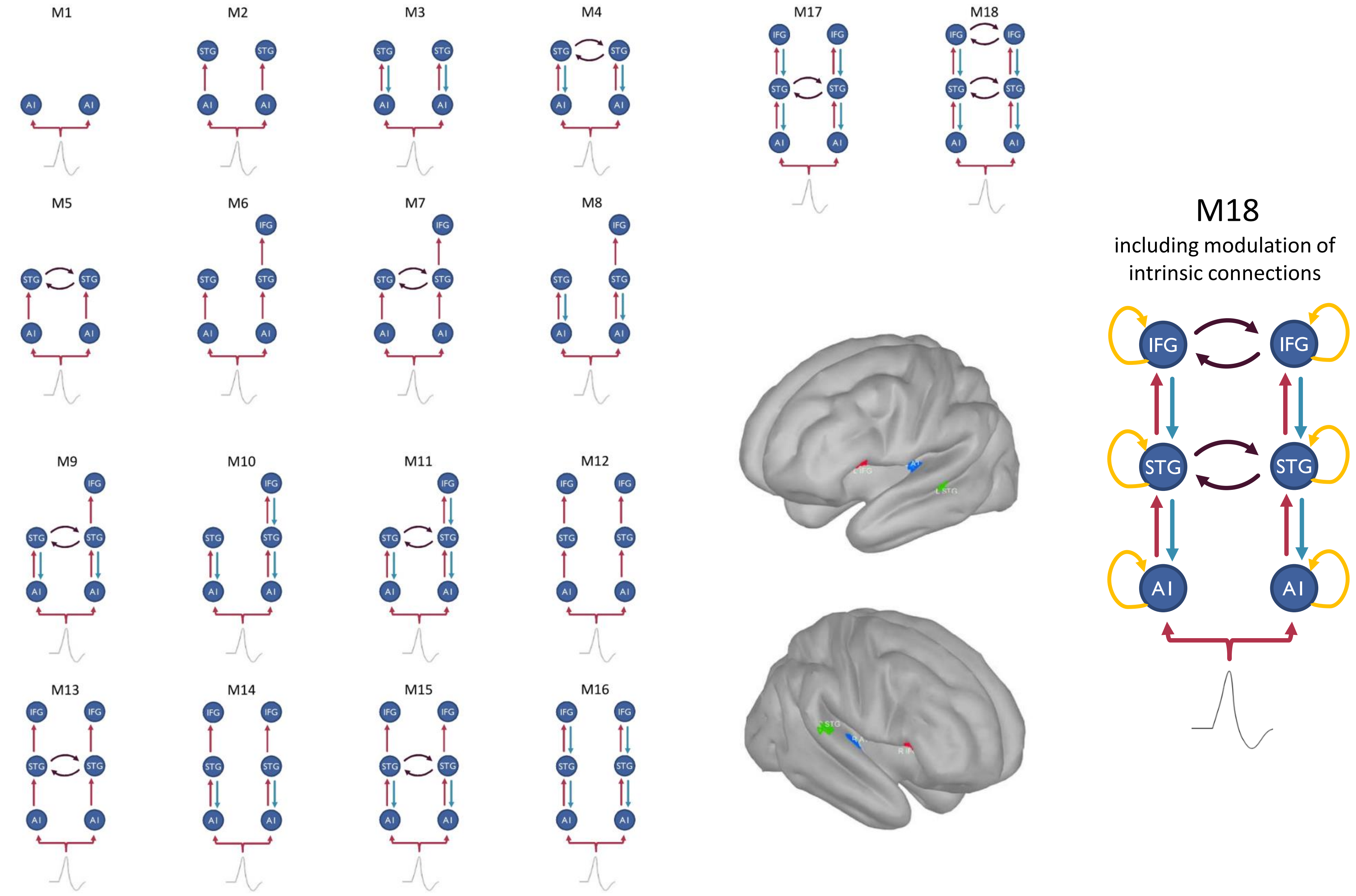

Supplementary Figure 6: Schematic display of 18 different models (M1->M18) used in dynamic causal modeling of deviant stimulus effects (MMN responses) in SPM12. Each model receives subcortical input at the A1 sources eliciting further transient perturbations in the remaining sources. The models incorporate a different number of sources/nodes (2, 4, 5, 6) as well as a different type and arrangement of connections (forward, feedback, lateral). Sources: In the left view of the brain **(1)** left A1 – in blue; **(2)** left IFG – in red; **(3)** left STG – in green; in the right view of the brain **(4)** right A2 – in blue; **(5)** right IFG – in red; **(6)** right STG – in green.

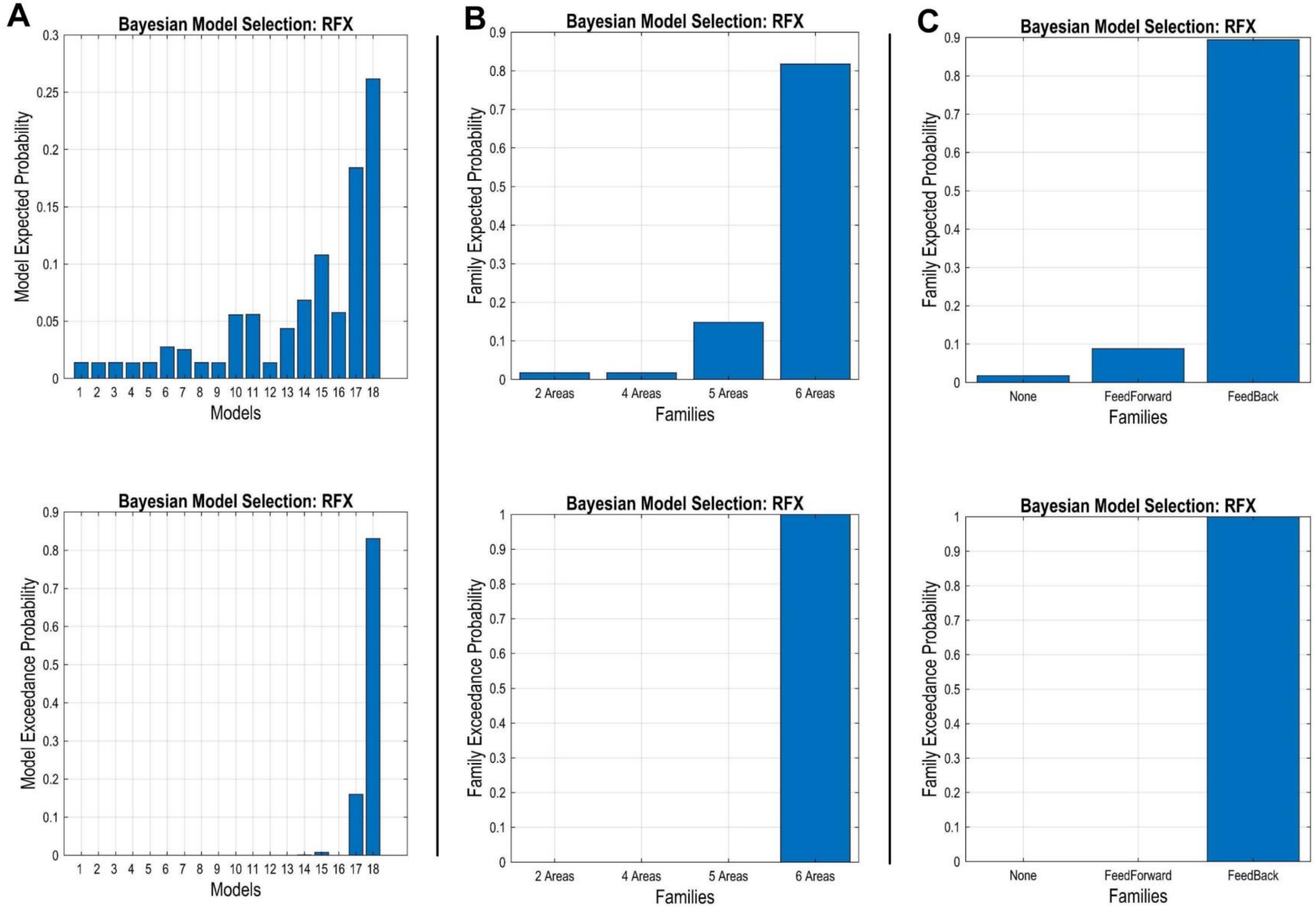

Supplementary Figure 7A: (A) Population-level best model resulting from a Bayesian model comparison : Random effects Bayesian model selection showed that Model #18 had the greatest evidence. (B) and (C): Family-wise Bayesian model selection showed that the best models included two frontal regions (bilateral) and the presence of both backward and forward connections, as well as lateral connections.

Note: On the top bar graphs is shown each model conventional posterior probability (i.e., the expected probability that the k-th model generated the data for a randomly selected subject). On the bottom bar graphs, exceedance probability is the probability of each model being better than any other model. The advantage of using exceedance probabilities is that they are sensitive to the confidence in the posterior probability and easily interpretable (since they sum to unity over all models tested). The best model is the one with highest exceedance probability (equivalently, the highest expected posterior probability; the ranking is the same).

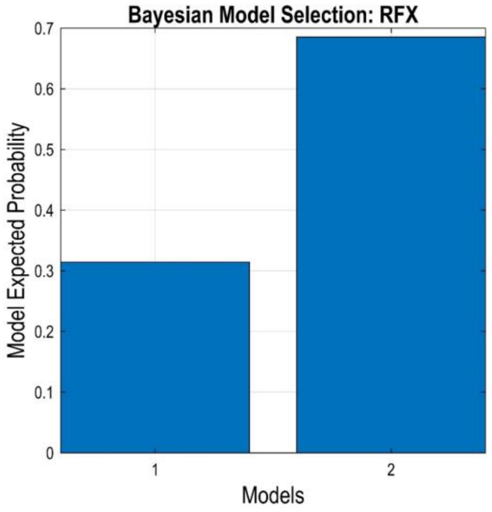

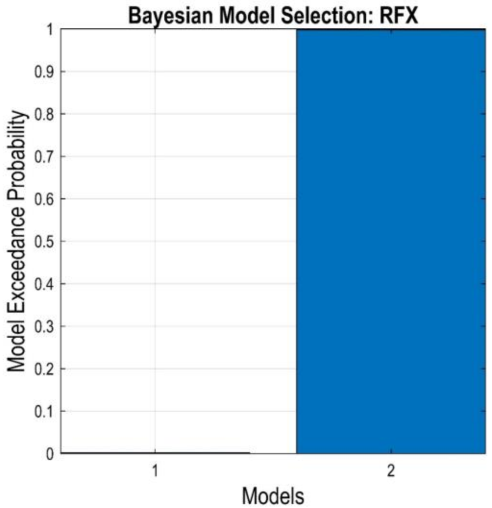

Supplementary Figure 7B: Population-level best model resulting from a Bayesian model comparison : Random effects Bayesian model selection showed that labeled Model #2 (representing here a fully-connected original M18 model *with modulation* of intrinsic/self connection at each node) had greater evidence compared to the labeled Model #1 (representing here a fully-connected original M18 model *without modulation* of intrinsic/self connection at each node), and was selected for subsequent quantitative analysis of effective connectivity across the two populations.
Note: On the top bar graph is shown each model conventional posterior probability (i.e., the expected probability that the k-th model generated the data for a randomly selected subject).
On the bottom bar graph, exceedance probability is the probability of each model being better than any other model. The advantage of using exceedance probabilities is that they are sensitive to the confidence in the posterior probability and easily interpretable (since they sum to unity over all models tested). The best model is the one with highest exceedance probability (equivalently, the highest expected posterior probability; the ranking is the same).

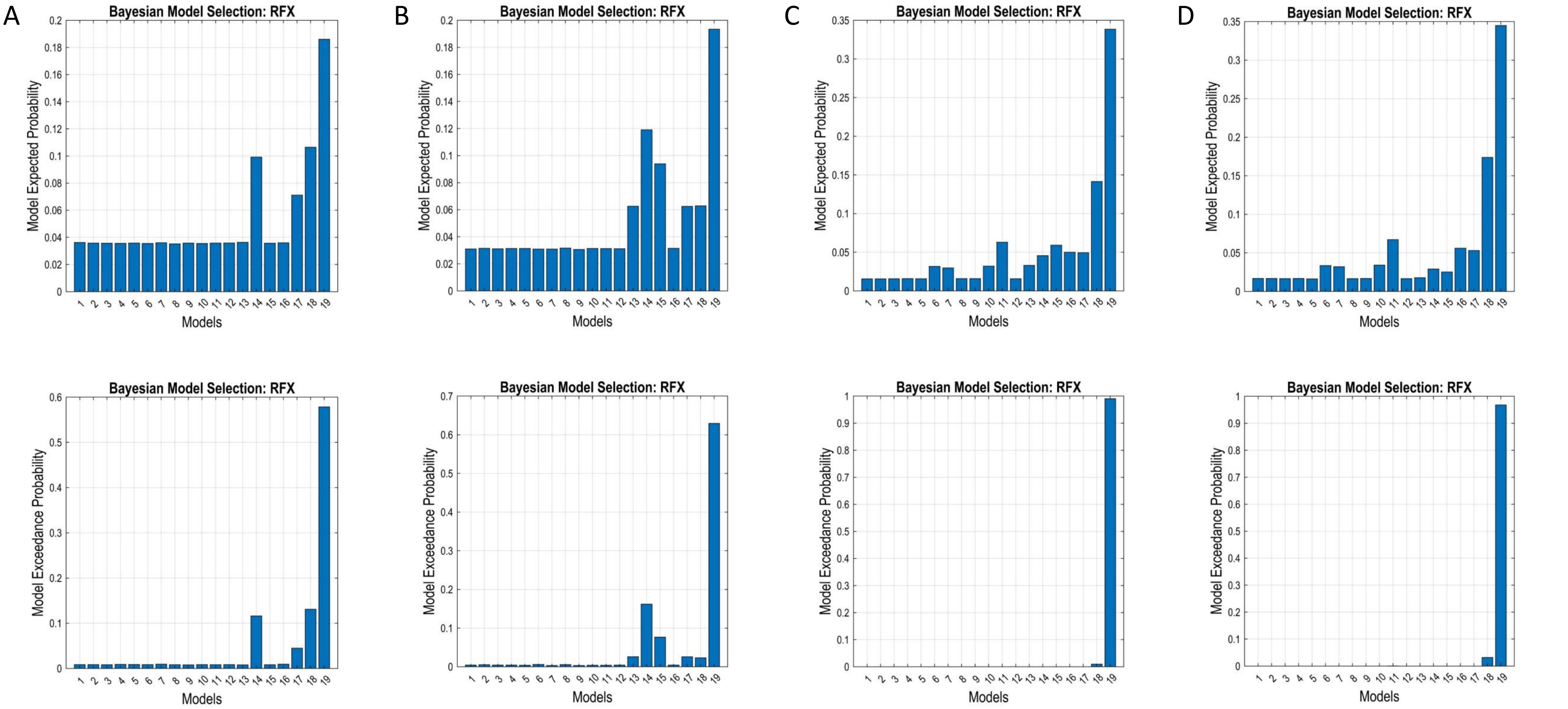

Supplementary Figure 7C: Population-level best model resulting from a Bayesian model comparison : Random effects Bayesian model selection showed that labeled Model #19 (representing here a fully-connected original M18 model *with modulation* of intrinsic/self connection at each node) had greater evidence compared to the other models, and was selected for subsequent quantitative analysis of effective connectivity across the two subgroups. Here, Bayesian model comparison was conducted separately for A) MK-6240+; B) PiB+; C) MK-6240-; D) PiB- subjects. Note: On the top bar graphs is shown each model conventional posterior probability (i.e., the expected probability that the k-th model generated the data for a randomly selected subject). On the bottom bar graphs, exceedance probability is the probability of each model being better than any other model. The advantage of using exceedance probabilities is that they are sensitive to the confidence in the posterior probability and easily interpretable (since they sum to unity over all models tested). The best model is the one with highest exceedance probability (equivalently, the highest expected posterior probability; the ranking is the same).

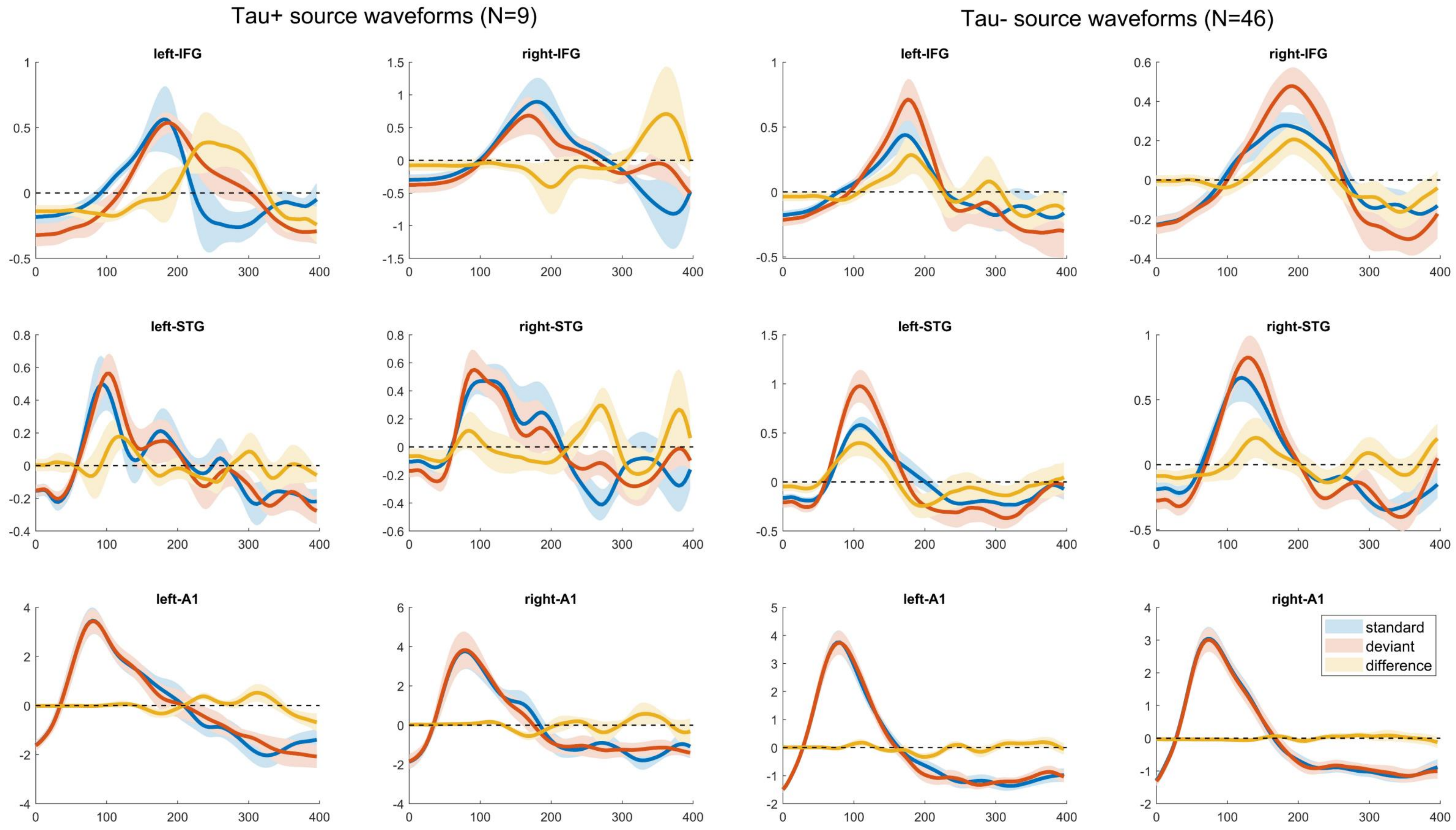

Supplementary Figure 8A: Grandaveraged source waveforms for standard, deviant and difference waves from data belonging to MK-6240+ and MK-6240- (as Tau+ and Tau-) subjects. The shaded areas display the standard error of the means.

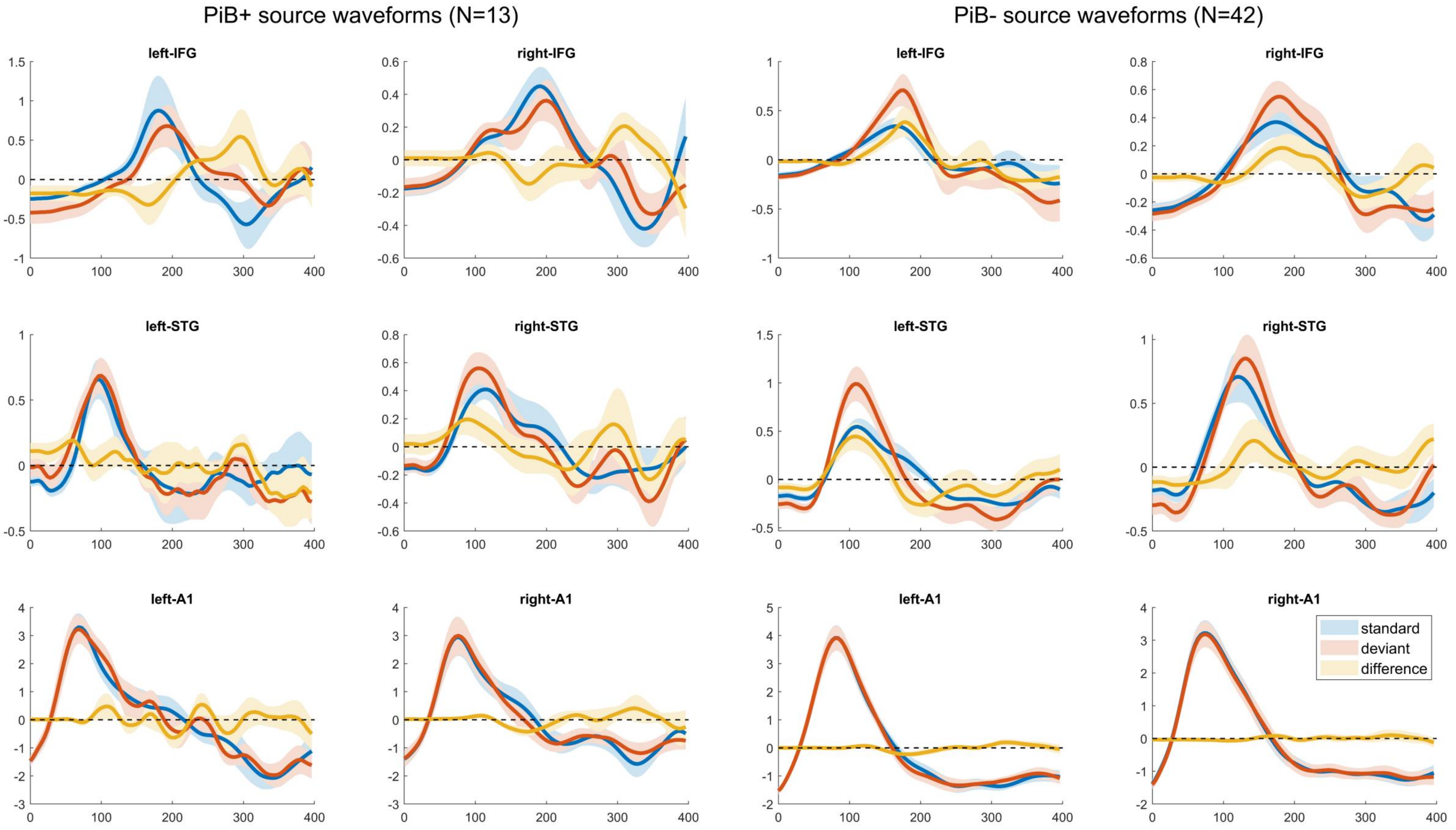

Supplementary Figure 8B: Grandaveraged source waveforms for standard, deviant and difference waves from data belonging to PiB+ and PiB- subjects. The shaded areas display the standard error of the means.

	PiB +<br>n = 13	PiB –<br>n = 43	Tau +<br>n = 9	Tau –<br>n = 46
**Sex**	Male = 31%<br>Female = 69%	Male = 35%<br>Female = 65%	Male = 44%<br>Female = 56%	Male = 33%<br>Female = 67%
**Age**<br>Median (IQR)	73.75 (70.5 – 75.34)	68.15 (62.64 – 72.48)	73.75 (73.50 – 75.34)	68.17 (62.72 – 72.5)
**Category Naming Score**<br>Median (IQR)	21 (18 – 25)	21 (18 – 24)	19 (17 – 24)	21 (18 – 25)
**Letter fluency**<br>Median (IQR)	42 (37 – 50)	42 (37 – 50.25)	40 (37 – 44)	42 (36 – 51)
**RAVLT Recognition True Positives**<br>Median (IQR)	13 (12 – 14)	14.5 (13 – 15)	12 (11 – 15)	14 (13 – 15)
**TMTA Errors**<br>Median (IQR)	0 (0 – 0)	0 (0 – 0)	0 (0 – 0)	0 (0 – 0)
**TMTA Time**<br>Median (IQR)	26 (19 – 31)	22 (19 – 27)	28 (22 - 31)	22 (19 – 27)
**TMTB Errors**<br>Median (IQR)	0 (0 – 0)	0 (0 – 1)	0 (0 – 1)	0 (0 – 1)
**TMTB Time**<br>Median (IQR)	61 (53 – 89)	61 (46 – 73.25)	72 (60 -89)	61 (46 – 75)

Supplementary Table 1: Demographic and neuropsychological test data.

	Tau +		Tau -	
	**PiB +**<br>n = 7	**PiB –**<br>n = 2	**PiB +**<br>n = 6	**PiB –**<br>n = 40
**Dementia**	1 (14%)	0 (0%)	0%	2 (5%)
**MCI**	2 (29%)	0 (0%)	0%	0 (0%)
**ADRC**	3 (43%)	0 (0%)	1 (17%)	9 (23%)
**WRAP**	4 (57%)	2 (100%)	5 (83%)	31 (77%)
**Sex**	Male = 3 (43%)<br>Female = 4 (57%)	Male = 1 (50%)<br>Female = 1 (50%)	Male = 1 (17%)<br>Female = 5 (83%)	Male = 14 (35%)<br>Female = 26 (65%)
**Age**<br>Median (IQR)	73.75 (73.50 – 74.80)	76.5 (75.04 – 77.96)	71.56 (66.96 – 75.30)	69.03 (62.47 – 72.44)
**Category Naming Score**<br>Median (IQR)	19 (17.50 – 23.50)	17.5 (14.25 – 20.75)	23 (19.50 – 27.25)	21.5 (19 – 25)
**Letter fluency**<br>Median (IQR)	43 (37 – 47)	39.5 (39.25 – 39.75)	41 (34 – 51)	44 (37 – 54.50)
**RAVLT Recognition True Positives**<br>Median (IQR)	12 (10.50 – 14)	13.5 (12.75 – 14.25)	13.5 (13 – 14)	15 (14 – 15)
**TMTA Errors**<br>Median (IQR)	0 (0 – 0)	0 (0 – 0)	0 (0 – 0)	0 (0 – 0)
**TMTA Time**<br>Median (IQR)	26 (21 – 28.50)	40 (37 – 43)	27.5 (18.25 – 39.75)	22 (19 – 26.25)
**TMTB Errors**<br>Median (IQR)	0 (0 – 0.50)	0.5 (0.25 – 0.75)	0 (0 – 0)	0 (0 – 1)
**TMTB Time**<br>Median (IQR)	61 (56.50 – 80.50)	93.5 (83.75 – 103.25)	67.5 (47.5 – 104)	58 (46 – 71.25)

Supplementary Table 2: Breakdown of demographic and neuropsychological test data according to combined Tau and PiB status.
